# Is Dexamethasone superior to Ketorolac in reducing pain, swelling and trismus following mandibular third molar removal? A split mouth triple-blind randomized clinical trial

**DOI:** 10.4317/medoral.24088

**Published:** 2020-11-28

**Authors:** Allan Vinícius Martins-de-Barros, Ana Maria Ipólito Barros, Anna Karolline Cadengue de Siqueira, Eudes Euler de Souza Lucena, Pedro Henrique Sette de Souza, Fábio Andrey da Costa Araújo

**Affiliations:** 1 DDS. Resident in Oral and Maxillofacial Surgery. Hospital Universitário Oswaldo Cruz, University of Pernambuco, Recife, Brazil; 2 DDS. Resident in Dentistry (emphasis in Oncology). Hospital do Câncer de Pernambuco, University of Pernambuco, Recife, Brazil; 3 DDS. School of Dentistry, University of Pernambuco, Campus Arcoverde, Arcoverde, Brazil; 4 DDS, MSc, PhD. Associate Professor. Multicampi Medical Science School, Federal University of Rio Grande do Norte, Natal, Brazil; 5 DDS, MSc, PhD. Associate Professor. School of Dentistry, University of Pernambuco, Campus Arcoverde, Arcoverde, Brazil

## Abstract

**Background:**

The preemptive use of anti-inflammatory drugs, such as corticosteroids and NSAIDs, has the potential to reduce pain, swelling and trismus following oral surgery. The aim of this study was to compare the efficacy of dexamethasone and ketorolac tromethamine in reducing pain, swelling and trismus after mandibular third molar removal.

**Material and Methods:**

The researches implemented a triple-blind, randomized clinical trial. The study was conducted with ASA I individuals aging between 18 and 35 years, which were randomized and submitted to two interventions, one with 8mg dexamethasone and the other with 20mg ketorolac tromethamine given 1h before the procedure. The primary predictor variable was the use of dexamethasone or ketorolac. The primary outcome variable was the postoperative pain level, measured with a Visual Analogue Scale. The secondary outcome variables were the amount of rescue analgesic consumed, swelling and trismus. Repeated-measures ANOVA and t-test for paired samples were used to compare the means. Significance was set at *p* < 0.05.

**Results:**

Fifty individuals were randomized and allocated to intervention, and the sample was composed of 40 subjects who completed the study (27 female and 13 male). Dexamethasone, when compared to ketorolac tromethamine, showed a significantly higher reduction in pain level at 8h, 16h, 24h, 32h, 40h and 72h, in swelling and trismus at 24h, 48h, 72h and 7 days and in total number of rescue analgesics taken up to 72h postoperative (*p* < 0.05).

**Conclusions:**

The clinical performance of dexamethasone in controlling pain, swelling and trismus after mandibular third molar removal was superior to ketorolac tromethamine’s.

** Key words:**Third Molar, anti-inflammatory agents, dexamethasone, ketorolac.

## Introduction

The inflammatory process triggered by tissue trauma from third molar surgery commonly results in pain, swelling and trismus, and is responsible for significant functional and aesthetic discomfort during the postoperative period (1,2).

In these cases, the preemptive use of anti-inflammatory drugs has the potential to reduce the intensity and morbidity of these events by inhibiting the inflammatory response prior to surgical trauma (3-6). Two classes of drugs commonly used for this purpose are corticosteroids and non-steroidal anti-inflammatory drugs (NSAIDs) (2,7-9).

Corticosteroids are drugs capable of suppressing the inflammatory response and immune function at various stages (10,11). The perioperative use of systemic corticosteroids is a common approach to control inflammatory events after third molars removal, with potential to reduce early and late swelling and trismus, although its effects on pain control are controversial (5). Dexamethasone is a long-acting corticosteroid, a synthetic analogue of prednisolone, which has a potent anti-inflammatory effect, mainly by promoting the synthesis of regulatory proteins of the inflammatory process, such as lipocortin and vasocortin (10-12).

NSAIDs are drugs which inhibit the Cyclooxygenase (COX) enzyme involved in the synthesis of chemical mediators of inflammation (13,14). NSAIDs are effective in the treatment of acute inflammatory conditions and are employed to decrease postoperative swelling and trismus, delay the onset and reduce the intensity of pain following oral surgery, with fewer undesirable adverse effects (2,7,9). Ketorolac tromethamine is a non-selective COX inhibitor and has important analgesic, anti-inflammatory and antipyretic properties (15).

Both dexamethasone and ketorolac tromethamine are used effectively in the field of oral surgery in various dosages and administration routes (6,12,15-17). However, currently available scientific literature is scarce and points out the need for methodologically adequate studies to compare these two drugs in order to elucidate their impact on reducing morbidity in the postoperative period and to adequately assess the optimal dosage and the proper time and route of administration for preemptive drugs in third molar surgery.

Thus, the objective of this study was to compare via clinical parameters the anti-inflammatory effect of 8mg dexamethasone orally and 20mg ketorolac tromethamine sublingually when administered prior to impacted mandibular third molars surgery. The specific aim of this study was to determine the differences in pain level, consumption of rescue analgesic, swelling and mouth opening limitation (trismus) between the two anti-inflammatory drugs. The investigators hypothesize that the preemptive administration of corticosteroids is more effective in controlling postoperative swelling and trismus, although the preemptive administration of NSAIDs is more effective in reducing postoperative pain.

## Material and Methods

- Study design

The investigators implemented a triple-blind randomized clinical trial design using the split mouth model. The primary predictor variable was the use of Dexamethasone or Ketorolac Tromethamine. The study was developed at the Dental Clinic of the University of Pernambuco, Arcoverde Campus, located in the Northeast of Brazil, between July 2017 and April 2018, approved by the Committee of Ethics of the University of Pernambuco, under protocol no. 1,952,038 and registry CAAE 63926316.5.0000.5207 and performed according to the Declaration of Helsinki, registered in the Brazilian Registry of Clinical Trials – ReBEC (UTN: U1111-1194-9558) and conducted according to the Consolidated Standards of Reporting Trials (CONSORT).

All participants were informed of the objectives of the research, as well as their risks and benefits, and those who agreed to participate in the study signed the Informed Consent Form (ICF).

- Study population

The study population was composed of adult individuals who were indicated for surgical removal of mandibular third molars with total or partial bone impaction. The inclusion criteria of the study were: individuals of both genders aging between 18 and 35 years, classified as ASA I according to the American Society of Anesthesiologists, and who have two mandibular third molars in similar positions according to the Pell and Gregory’s and Winter’s classifications. To reduce the risk of classification bias, the assessment of tooth position was performed by a single subject expert PhD in Oral and Maxillofacial Surgery in digital panoramic radiographs.

Considering its influence on the surgical procedure and/or on the evaluated outcome variables, the exclusion criteria for the study included: those individuals who were allergic to any of the drugs used in the study; who presented a disease or systemic condition that contraindicated the procedure; who were in gestation or lactation period; who used chronic medications or other substances that influence the inflammatory response; who presented symptomatology compatible with local infection at the time of the procedure; those for which the total surgical procedure time was equal to or greater than 40 minutes (7); who refused to adopt the drug regimen proposed by the study; or who refused to participate in the data collection steps.

- Randomization and blinding methods

By implementing the split mouth model, each participant underwent two surgical procedures, with wash-out interval of at least three weeks between them (8). Prior to the first procedure, randomization of the first side to be operated (right or left) was performed using the “heads or tails” technique using a coin, where the head represented the right side and tails the left side.

The drug randomization was performed with the assistance of the simple randomization service from Sealed Envelope™ (available on: https://www.sealedenvelope.com/). After the assessment for eligibility, each participant was assigned to a random code (A or B), which determined the combination of Tablets that were administered in the waiting room of the Dental Clinic, one hour before the first surgical procedure, by an external collaborator not involved in any of the data collection or analysis stages. In this phase, we assured that half of the subjects randomly received the code A and the other half received the code B.

The combination of Tablets to be administered in the interventions were coded in A and B to guarantee the blinding of the study. The code A represented a combination of 8mg oral dexamethasone (Aché Laboratórios Farmacêuticos SA, Guarulhos, SP, Brazil) + sublingual placebo. Thus, the code B represented oral placebo + 20mg sublingual ketorolac tromethamine (EMS Sigma Pharma Ltda., Hortolândia/SP, Brazil). The Tablets’ composition in combinations A and B was kept in secret and only revealed after completing the full statistical analysis of the data.

The placebo Tablets were handled by the Pharmacy School of the Federal University of Rio Grande do Norte (Natal/RN, Brazil) in the same form, color and size as the reference Tablets with active principle and were only used to guarantee the participants’ blinding, given the difference between the administration routes of these drugs.

The second surgical procedure was performed on the contralateral side of the first procedure after the wash-out period (7), with administration of the other combination of Tablets.

For the blinding of this Clinical Trial, the participants, the surgeon, the examiner and the statistician did not know the drug used in each intervention, thus providing a triple blinding in the study.

- Surgical technique

The participants were submitted to a previous dental consultation, where their sociodemographic characteristics, vital signs, medical history and position of third mandibular molars were evaluated and recorded in a specific file.

The surgical procedures were performed by an expert Oral and Maxillofacial Surgeon and started one hour after administrating the anti-inflammatory drug. All participants prophylactically received 1g of amoxicillin orally and the antisepsis and aseptic chain maintenance protocol were performed.

Local anesthesia of the inferior alveolar, lingual and buccal nerves was performed using an anesthetic solution of 2% mepivacaine with 1:100,000 adrenaline (DLA Pharmaceutical Ltda., Catanduva/SP, Brazil). The surgical procedure to extract the impacted third molar was performed with an incision in the distal region of the gingival sulcus of the second molar and a side oblique incision directed posteriorly towards the mandibular ramus, and then the mucoperiosteal flap was raised. Osteotomies and odontosection were performed with hand-pieces and carbide burs No. 702 (Dentsply International, New York, EUA) under sterile saline solution irrigation according to the condition of the tooth impaction. Elevators and forceps were used when necessary. The primary closure of the wound was performed with 3-0 silk threads. At the end, direct compression with sterile gauze was kept on the spot for 30 minutes. The duration of the procedure was timed, considering the interval between beginning the first incision and the end of the last suture, and recorded in minutes at the end of each procedure.

After the surgical procedure, each participant received postoperative instructions and 10 Tablets of 500mg dipyrone monohydrate (EMS Sigma Pharma Ltda., Hortolândia/SP, Brazil) and were instructed to use them as an oral rescue analgesic if there was any need for pain relief, with a minimum interval of six hours between doses.

- Evaluation of outcome variables

The primary outcome variable was postoperative pain level. The secondary outcome variables were the amount of rescue analgesic consumption, swelling and trismus. All outcome variables were clinically and prospectively evaluated during the study.

Postoperative pain level was measured using the Visual Analogue Scale (VAS) containing color parameters, face scales and pain categorization ranging from 0 (absence of pain) to 10 (severe pain). Subjects were instructed to measure pain according to VAS before and immediately after the surgical procedure, as well as at 8-hour intervals during the first 72 hours and on the 7th postoperative day. The total number of rescue analgesic Tablets consumed postoperatively up to the first 72 hours and the exact moment of consumption of each Tablet were also recorded.

Swelling and trismus were measured by a blinded and previously-trained examiner and the measurements were performed prior to the surgical procedure (baseline) in the immediate postoperative period (0h), and at intervals of 24h, 48h, 72h and 7 days after surgery.

**Figure 1:**
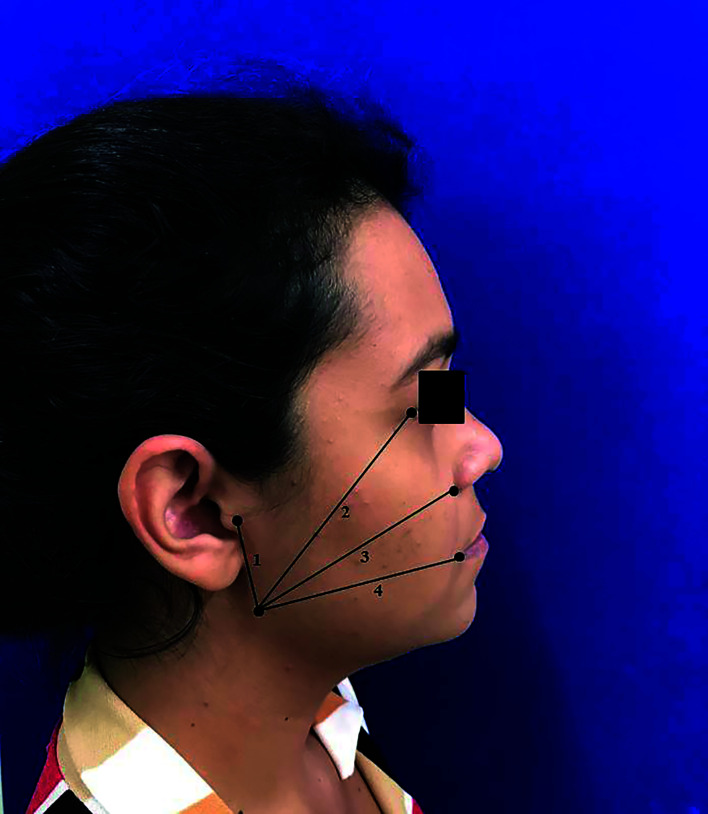
Reference points on the subject’s face and linear demarcations for swelling measurement. 1. AnM-Tr; 2. AnM-LPC; 3. AnM-NW; 4. AnM-LC.

Swelling was evaluated by measuring reference points on the subject’s face with a tape measure. The following reference lines were measured as described by Laureano Filho *et al*. (16): 1) The distance from angle of the mandible to the ear tragus (AnM-Tr); 2) The distance from angle of the mandible to the lateral palpebral commissure (AnM-LPC); 3) The distance from angle of the mandible to the nose wing (AnM-NW); 4) The distance from angle of the mandible to the labial commissure (AnM-LC) (Fig. 1). The swelling quantification was performed through the increase percentage in the measurements obtained in the postoperative period in relation to the baseline measurements. The anatomical reference points were identified with permanent marker brush to guarantee reproducing the measurement technique during the evaluation period.

Trismus was evaluated based on the mouth opening limitation. The interincisal distance (in millimeters) during maximum mouth opening without pain was measured with a calibrated digital caliper with reference to the incisal edge of the upper and lower left central incisors (7) and the mouth opening limitation was determined by the difference in millimeters between the preoperative baseline interincisal distance value and the values obtained in the postoperative period.

- Statistical methods and analysis

The sample size calculation was performed in the OpenEpi Version 3.01 statistical software through the mean differences of the primary outcome variable between the study groups in a pilot study (mean difference set as 1.5, with standard deviation of 1.94 and 2.76 for groups A and B, respectively), establishing a 95% confidence interval and 80% power, with type I error of 0.05. A sample of 40 surgical procedures for each group was determined.

The database of this study was built on IBM SPSS® software version 20.0 platform (IBM Corp., Armonk, NY, USA). This process was performed through double-tabulation to minimize the risk of typing error, where two different typists typed the same files independently. The statistical testing phase began upon verification of agreement between the two databases. The Kolmogorov-Smirnov test was used to test the normality of the variables. As all of the variables showed normal distribution, the means were compared using repeated-measures ANOVA and t-test for paired samples. A significance level of 5% (*p* < 0.05) was adopted for all statistical tests.

## Results

**Figure 2:**
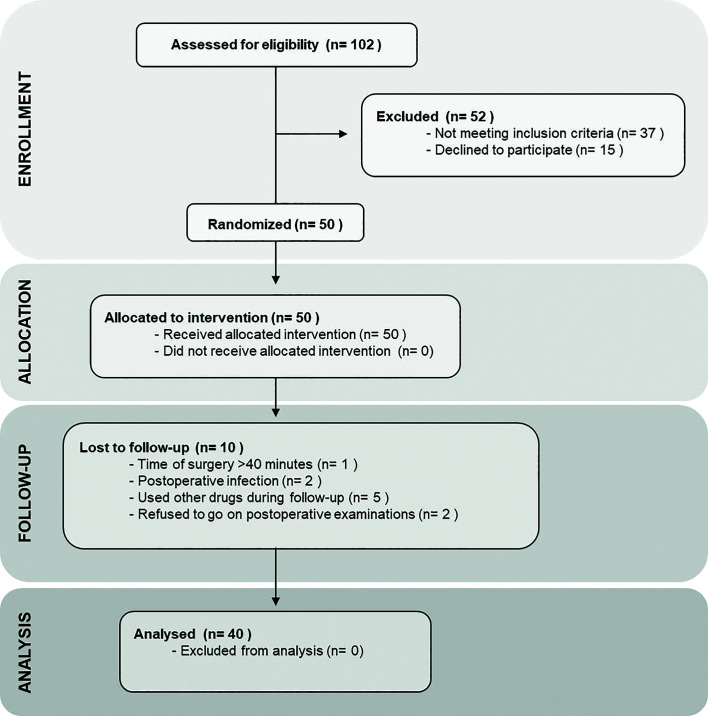
Flow diagram of the participants according to CONSORT statement.

During the recruitment phase, 102 individuals potentially eligible for the study were screened. Of these, 52 were excluded because they did not meet the inclusion criteria, and therefore 50 participants were enrolled and allocated to the interventions. According to the exclusion criteria, there were 10 participants lost to follow-up, and thus the data obtained from 40 individuals that completed the study were used in the statistical analysis phase, as shown in the CONSORT Flowchart (Fig. 2). The final sample of the study was composed by 27 women and 13 men, with age ranging between 18 and 27 years, with a mean of 22,05 ± 2,47 years. Regarding to ethnic background, the participants were mostly white (n = 21) or brown (n = 18), and only one subject was black. Clinical data of the participants and the degree of impaction and the position of the mandibular third molars according to Pell and Gregory and Winter classifications are described in Table 1.

**Table 1:**
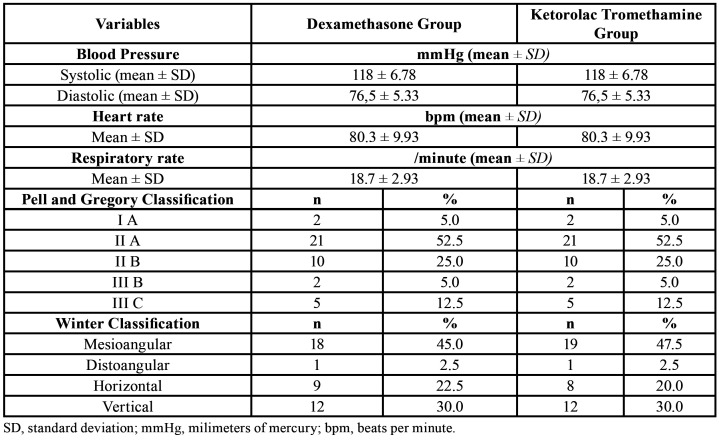
Baseline clinical characteristics of the participants in dexamethasone and ketorolac tromethamine groups.

The surgical procedure had an average duration of 20.75 ± 8.3 min. in the dexamethasone group, and 20.97 ± 7.4 min. in the ketorolac tromethamine group, and this difference was not statistically significant (*p* = 0.880). During the intervention with dexamethasone, 32 (80%) individuals were submitted to osteotomy and 22 (55%) to tooth sectioning, while in the intervention with ketorolac tromethamine, osteotomy was performed in 29 (72.5%) individuals, and tooth sectioning in 23 (57.5%).

When submitted to dexamethasone administration, participants had lower levels of pain at all evaluation times in the first 72 postoperative hours. A statistically significant difference was observed in the periods of 8h, 16h, 24h, 32h, 40h and 72h. The comparison between the averages of pain levels presented in the postoperative period for dexamethasone and ketorolac tromethamine is shown in Table 2.

In the dexamethasone group, 40% (n = 16) of the participants did not consume any of the rescue analgesic Tablets, whereas this number was 27.5% (n = 11) for ketorolac tromethamine. The average consumption of rescue analgesic Tablets by up to 72 hours with the ketorolac tromethamine administration (2.02 ± 1.94) was higher than the mean value observed with dexamethasone (1.30 ± 1.53), with *p* = 0.026. In addition, the mean time to ingest the first rescue analgesic Tablet was slightly lower with ketorolac tromethamine (10.02 ± 9.05) when compared to dexamethasone (10.65 ± 12.27), however there was no statistical significance in this result (*p* = 0.558).

Table 3 shows the measures comparison of swelling and trismus during the postoperative period. Dexamethasone presented better performance in relation to the swelling and trismus means when compared to ketorolac tromethamine. The results were statistically significant (*p* ≤ 0.01) for the variables “distance AnM-NW”, “distance AnM-LC” and “limitation of mouth opening” at 24h, 48h, 72h and 7 days.

**Figure 3:**
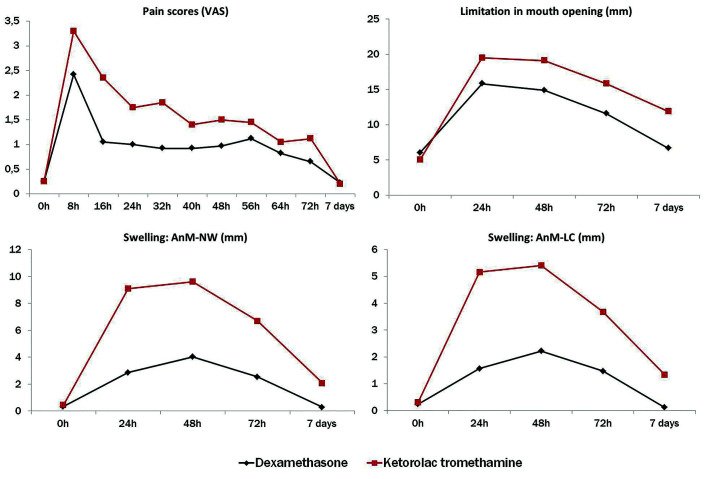
Distribution of estimated means of pain, swelling and limitation of mouth opening for dexamethasone and ketorolac tromethamine groups over the time.

Repeated-measures ANOVA showed that the means of pain (F = 3.341 [5.70 – 222.43], *p* = 0.004), swelling (AnM-NW: F = 24.36 [3.20 – 125.08], *p* < 0.001; AnM-LC: F = 24.35 [1.97 – 77.11], *p* < 0.001) and limitation of mouth opening (F = 4.782 [2.60 – 101.44], *p* = 0.006) differed significantly in function of the follow-up time, favoring the group dexamethasone over the group ketorolac in all these variables. Fig. 3 shows the distribution of estimated means of pain, swelling and limitation of mouth opening over the time.

No adverse events, accidents, or pre-, trans- or postoperative complications related directly or indirectly to the use of the drugs under study were observed.

**Table 2:**
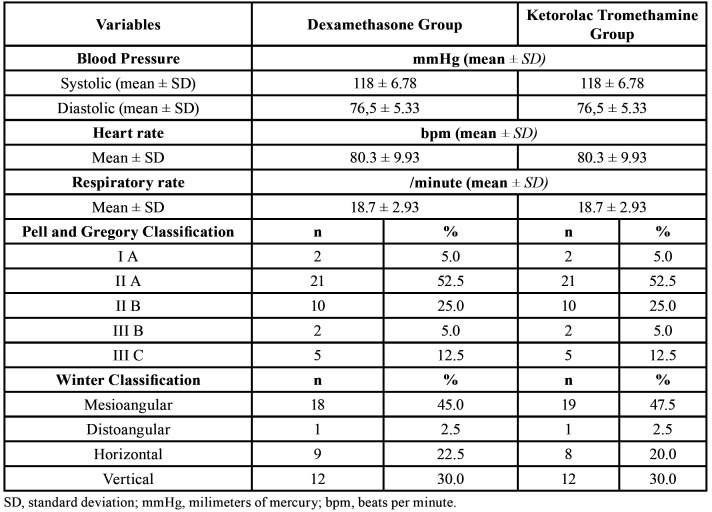
Comparison of pain levels between dexamethasone and ketorolac tromethamine groups in different moments of postoperative period.

**Table 3:**
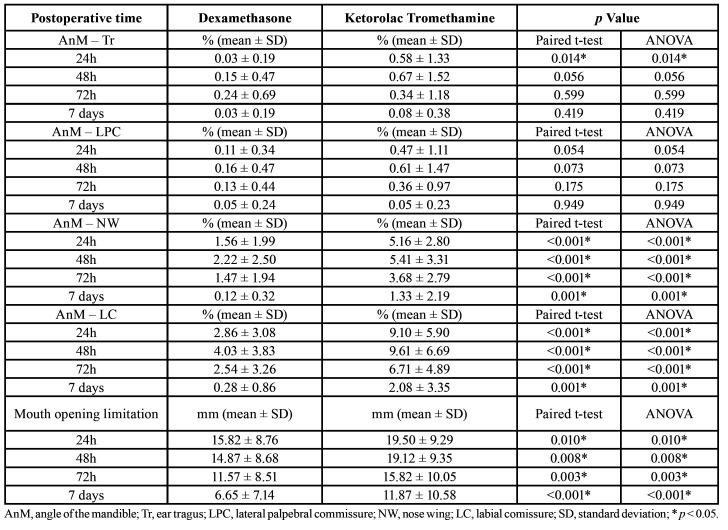
Comparison of swelling and trismus (mouth opening limitation) between dexamethasone and ketorolac tromethamine groups in different moments of postoperative period.

## Discussion

The purpose of this study was to assess the anti-inflammatory efficacy of a single dose of 8mg dexamethasone orally when compared to a single dose of 20mg ketorolac tromethamine sublingually when administered one hour prior to impacted third molar surgery. This study refuted the hypothesis that the preemptive administration of NSAIDs is more effective in reducing postoperative pain, as Dexamethasone showed better results in controlling pain, swelling and trismus following impacted third molar surgery when compared to ketorolac tromethamine. Several studies demonstrating the anti-inflammatory efficacy of dexamethasone and ketorolac tromethamine alone are available in the literature (6,15-20). However, this is the first study to compare the effect of these two drugs through the clinical parameters of pain, swelling and trismus when administered in a single preoperative dose.

Moreover, this study assesses the anti-inflammatory effects of preoperative sublingual ketorolac in impacted third molar surgery, when most available studies focused on the intravenous route (6,19,20). Although the administration of an initial dose of ketorolac tromethamine via the sublingual route is considered an off label practice in the U.S.A., in some countries, such as Brazil, it is only available in sublingual or injecTable formulations (21). In view of that, the sublingual route can be considered a self-administered, safe and more comforTable alternative to parenteral routes, with little clinical research on its efficacy in the field of oral surgery (15,21).

Postoperative pain is modulated by the release of chemical mediators of inflammation, which act to sensitize the peripheral nociceptors at the injury site, resulting in hyperalgesia (14,22,23). Studies using microdialysis found a directly proportional correlation between pain intensity after mandibular third molar surgery and Prostaglandin E2 (PGE2), Thromboxane B2 (TxB2) and Bradykinin levels at the surgical site (14,22,24). The role of corticosteroids in managing postoperative pain is still not fully understood (5,7,16,18). In these cases, the reduced swelling together with depression of bradykinin and histamine levels seems to contribute to pain reduction (10,11,23,24). Although some studies show a poor analgesic effect (12,16,18,22) associated with an insufficient reduction in local PGE2 levels with dexamethasone use (22,25), in the present study this corticosteroid was shown to be better than ketorolac tromethamine in reducing postoperative pain and the use of rescue analgesics.

Available studies point to the efficacy of ketorolac tromethamine in controlling moderate to severe pain, with effective reduction of PGE2 and TxB2 levels in the first hours of the postoperative period. However, none of these studies evaluated their analgesic effect in a single dose for a period greater than 12h (6,14,17,19,20,22). The results found by Ong *et al*. (6) for this drug suggest short lasting action with a limited long-term effect, which may be justified by its short half-life of approximately 5h (15). This factor may justify its worse performance in the present study, considering the 8h interval between the immediate postoperative period and the second pain assessment. Additionally, the reduced mean time to ingest the first rescue analgesic medication observed in kerotolac group, although not statiscally significant, could be related to the duration of its half-life. Similarly, in a systematic review, Costa *et al*. (4) evidenced the limited potential of other NSAIDs in preemptive analgesia.

Other clinical trials comparing the analgesic effect of NSAIDs and corticosteroids in several pharmacological protocols showed statistically similar results for both classes (26-28) or higher for corticosteroids (29), in agreement with the results obtained in this study.

Although several articles address the evaluation of postoperative pain with using ketorolac tromethamine in various dosages and administration routes (6,17,19,20), few studies have also evaluated the evolution of swelling and trismus in this period (28), while the effect of dexamethasone and other corticosteroids in these outcomes is better documented (12,16,18).

The formation of swelling after surgical trauma is mainly related to the vascular events of the inflammatory process, which promote vasodilation and increase vascular permeability, with consequent change in the local hydrostatic and osmotic pressures (23). The expected peak for swelling usually occurs between 48h and 72h after the procedure (7,8,12,16,18,27,29), similar results to those shown in the present study.

Corticosteroids are more effective than NSAIDs in controlling swelling (5,10,11). This is probably due to the combined action of lipocortin promotion, which acts on the inhibition of Phospholipase A2, and vasocortin, a histamine release inhibitor, which provide wider suppression of the inflammatory process, in addition to inhibiting the synthesis of eicosanoids (10,11).

Corticosteroids with intermediate action duration did not present a significant difference in reducing swelling when compared to NSAIDs (27). However, dexamethasone, which has longer lasting action, provides greater reduction of swelling even when compared to methylprednisolone (18). In this sense, the long-term anti-inflammatory action seems to play an important role in control of swelling in view of its late development in acute inflammation.

In a clinical trial comparing dexamethasone in a single preoperative dose and ketorolac tromethamine administered continuously until the second postoperative day, Oliveira *et al*. (28) did not observe a significant difference in pain evaluation and swelling, which reinforces the hypothesis that the deficit in the anti-inflammatory effect of ketorolac tromethamine in this study is related to its short half-life, about ten times lower than that of dexamethasone.

A wide variation in swelling assessment methods is observed in the literature, and the distance between anatomical points on the face is the most used for its cost-benefit and practicality (9,16,27). In the present study, the variation in the distance AnM-Tr and AnM-LPC due to swelling was not statistically significant. This was due to the fact that the anatomical location of the swelling after mandibular third molar surgeries did not significantly affect these regions.

Trismus, characterized by limited buccal opening, is a common complication after oral surgeries, with negative impact on daily functions and quality of life (1,2). The evaluation of postoperative trismus through interincisal distance is a well-established and widely used method (5). There is evidence that corticosteroids are more effective in controlling trismus when compared to NSAIDs (5,27,28), which supports the results found in the present study. Reducing trismus is thought to be directly proportional to the reduction of pain and swelling, although the exact mechanism by which this process occurs has not yet been fully elucidated (7,18,30).

Although adverse reactions are well described for both dexamethasone and ketorolac tromethamine (2,15), none were observed during the course of this study. Both drugs can safely be used in ASA I subjects undergoing oral surgery.

Regarding to study limitations, researchers sought to reduce the risk of bias by controlling for confounding variables such as age, surgical time, and impacted tooth position, which were similar in both groups, as shown in results. The inter-individual variables were controlled using the split mouth model, where each participant functioned as their own control, however minor variation in tooth position and degree of impaction between the right and left sides of the same individual could have been a potential confounder. In an attempt to control other factors that could influence the evaluated inflammatory outcomes, cases where the procedure lasted longer than 40 minutes, use of other drugs during the follow-up period and postoperative infection were excluded from this analysis (per-protocol analysis), as stated in exclusion criteria and supported by several similar studies (3,7,8,12,19,28).

The major limitation of this study is related to the assessment of postoperative pain. The level of pain was recorded in a self-reported file, which could lead to some inaccuracy and inter-individual variation in data recording; however, the V.A.S method to assess pain is widely used and accepted in scientific investigation. Moreover, the results could have been more elucidative if the pain levels in early postoperative period (< 8 hours) were assessed in short intervals to allow evaluation of short-term analgesic effect of both drugs.

The main strength of this research was its design that consisted in a triple-blinded randomized controlled clinical trial and included a prospective evaluation with multiple anti-inflammatory outcomes assessment during a clinically relevant postoperative period.

In conclusion, the clinical performance of dexamethasone in controlling postoperative inflammatory events was superior to that of ketorolac tromethamine in all evaluated parameters, which seems to be related to the particularities of its broad anti-inflammatory action mechanism and prolonged half-life. Thus, it is a more accessible and suiTable option for preemptive administration in oral surgery.
